# Smartphone Application Available for Extension Ladder Safety

**Published:** 2013-12-20

Falls from ladders are a common cause of preventable injuries. Misjudging the ladder angle is a significant risk factor for a fall. A free smartphone application is available from CDC to improve the safety of extension ladder users.

The ladder safety application features an indicator that provides visual, sound, and vibration signals to assist users in quickly positioning an extension ladder at an optimal angle. The application also provides graphic-oriented interactive reference materials, safety guidelines, and checklists for extension ladder selection, inspection, accessorizing, and use, and can serve as a convenient training tool.

CDC’s National Institute for Occupational Safety and Health developed the ladder safety application using innovative research, existing information from safety regulations and consensus standards, and input from industry. The application is available for Apple iPhone/iPad and Google Android devices, and is also available in Spanish. The ladder safety application and additional information are available at http://www.cdc.gov/niosh/topics/falls.

